# Two New Species of the Enigmatic *Leptokoenenia* (Eukoeneniidae: Palpigradi) from Brazil: First Record of the Genus Outside Intertidal Environments

**DOI:** 10.1371/journal.pone.0077840

**Published:** 2013-11-04

**Authors:** Maysa Fernanda Villela Rezende Souza, Rodrigo Lopes Ferreira

**Affiliations:** 1 Programa de Pós-graduação em Ecologia Aplicada da Universidade Federal de Lavras, Lavras, Minas Gerais, Brazil; 2 Laboratório de Ecologia Subterrânea, Setor de Zoologia, Departamento de Biologia, Universidade Federal de Lavras, Lavras, Minas Gerais, Brazil; Australian Museum, Australia

## Abstract

Two new species of Leptokoenenia Condé 1965 (Eukoeneniidae: Palpigradi), *Leptokoenenia pelada* sp. nov. and *Leptokoenenia thalassophobica* sp. nov., are described based on both male and female specimens collected in iron ore caves from the Brazilian Amazon. This is the first record of the genus for the Americas. Furthermore, a new combination is stated for *Eukoenenia gallii* based on its morphology.

## Introduction

The order Palpigradi is currently represented by six genera [1]. Eukoenenia Börner, 1901 is the most widely distributed genus and comprises approximately 80% of the described species. The genus Prokoenenia Börner, 1901 features six species that are distributed in the Americas and Southeast Asia and Koeneniodes Silvestri, 1913 is represented by eight species occurring in Asia and Africa. In contrast, *Triadokoenenia* (Rémy, 1950), Allokoenenia Silvestri, 1913 and Leptokoenenia Condé, 1965 are particularly poorly known: *Triadokoenenia* and *Allokoenenia* are monotypic genera (*T. millotorum* Remy, 1950 and A. afra Silvestri, 1913), found only in Madagascar and Guinea, respectively. *Leptokoenenia* is represented by two species *L. scurra* Monniot, 1966 (Congo) and *L. gerlachi* Condé, 1965 (Saudi Arabia), both found in coastal systems [1]. 

On the American continent, besides the genus *Eukoenenia*, there are only representatives of the genus *Prokoenenia* found in Chile and the United States [2–4]. In Brazil, until now, the order has been known from by eight species of *Eukoenenia* [5–12]. However, samples collected in ferruginous caves present in two areas in the state of Pará (Brazil), known as Serra Leste and Morro II, revealed the presence of Palpigradi of the genus *Leptokoenenia*. Therefore in this study, we describe two new species of this genus, which were found, however, in an environment quite different from that occupied by the two described species. 

According to Rowland & Sissom [13], the genus *Leptokoenenia* can be diagnosed by the following features: abdominal segment IX slightly wider than segment XI and approximately equal to the width of Segment VIII; pygidium slightly narrowed posteriorly and flagellum shorter than the opisthosoma. All these features are observed in an Italian species called *Eukoenenia gallii*, Christian 2009, which accordingly, fits perfectly in *Leptokoenenia*. Moreover, the flagellum of *E. gallii* is similar to those observed in all other *Leptokoenenia* species, being very distinct from those observed in *Eukoenenia* species. These observations suggest that the Italian species should be transferred to the genus *Leptokoenenia*, setting up a new combination. Flagellum shape appears to be an important character in definition of the genus within Eukoeneniidade. Therefore, it is highly recommended that the flagellum be described whenever possible. 

## Material and Methods

### Specimens and Laboratory Procedures

Field collecting permits were issued to R.L. Ferreira by SISBIO/CECAV (license no. 02001.003869/2006). Cave geographic coordinates were obtained through CECAV’s database (http://www4.icmbio.gov.br/cecav/index.php).

The specimens were found under rocks inside iron ore caves and were captured with a fine brush and placed in vials containing 70% ethanol.

 The specimens were examined by clearing in Nesbit’s solution and mounting in Hoyer’s medium on 2.6 x 7.6 cm glass slides using standard procedures developed for mites [14]. They were identified to genus level according to Rowland & Sissom [13]. All measurements are presented in micrometers (μm) and were taken using an ocular micrometer with a phase contrast microscope. Body length was measured from the apex of the propeltidium to the posterior margin of the opisthosoma.

 The following abbreviations were utilized, based on Barranco & Mayoral [15]: L, total body length (without flagellum); B, dorsal shield length; P, pedipalpus; I and IV, legs I and IV; ti, tibia; bta1, basitarsus 1; bta2, basitarsus 2; bta3, basitarsus 3; bta4, basitarsus 4; ta1, tarsus 1; ta2, tarsus 2; ta3, tarsus 3; a, width of basitarsus IV at level of seta r; er, distance between base of basitarsus IV and insertion of seta r; grt, tergal seta length; gla, lateral seta length; r, stiff seta length; t/r, ratio between length of basitarsus IV and stiff seta length; t/er, ratio between basitarsus IV length and distance to insertion of stiff seta; gla/grt, ratio between lengths of lateral and tergal setae; B/bta, ratio between lengths of prosomal shield and basitarsus IV; bta/ti, ratio between lengths of basitarsus IV and tibia IV. Setal nomenclature follows that of Condé [16–18].

The specimens are housed in the Coleção de Invertebrados Subterrâneos de Lavras, Departamento de Biologia, Universidade Federal de Lavras, Lavras, Minas Gerais (ISLA).

### Nomenclatural Acts

The electronic edition of this article conforms to the requirements of the amended International Code of Zoological Nomenclature, and hence the new names contained herein are available under that Code from the electronic edition of this article. This published work and the nomenclatural acts it contains have been registered in ZooBank, the online registration system for the ICZN. The ZooBank LSIDs (Life Science Identifiers) can be resolved and the associated information viewed through any standard web browser by appending the LSID to the prefix "http://zoobank.org/". The LSID for this publication is: urn:lsid:zoobank.org:pub:C48FC5E0-E453-4564-9E6A-E8CA6619FB90. The electronic edition of this work was published in a journal with an ISSN, and has been archived and is available from the following digital repositories: PubMed Central, LOCKSS.

## Results

### Taxonomic treatment

#### *Leptokoenenia* pelada sp. nov.

**(urn:lsid:zoobank.org:act:A995DB68-D0BD-492E-9E11-8A4A5BCCD417**)

 (Figures 1 - 9)

**Figure 1:**
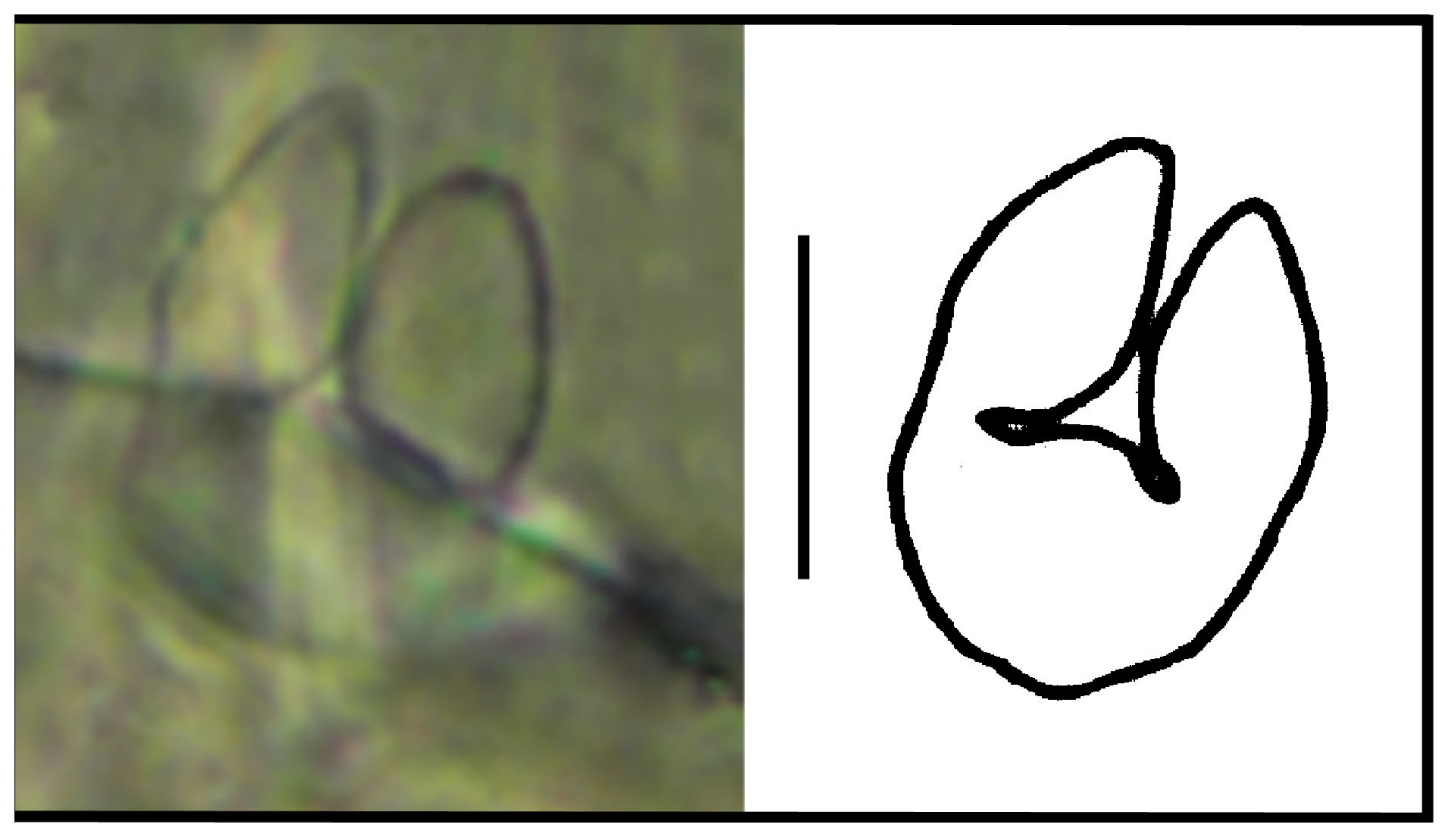
*Leptokoenenia pelada* sp. nov. Frontal organ of female 2. Scale bar 10 µm.

**Figure 2:**
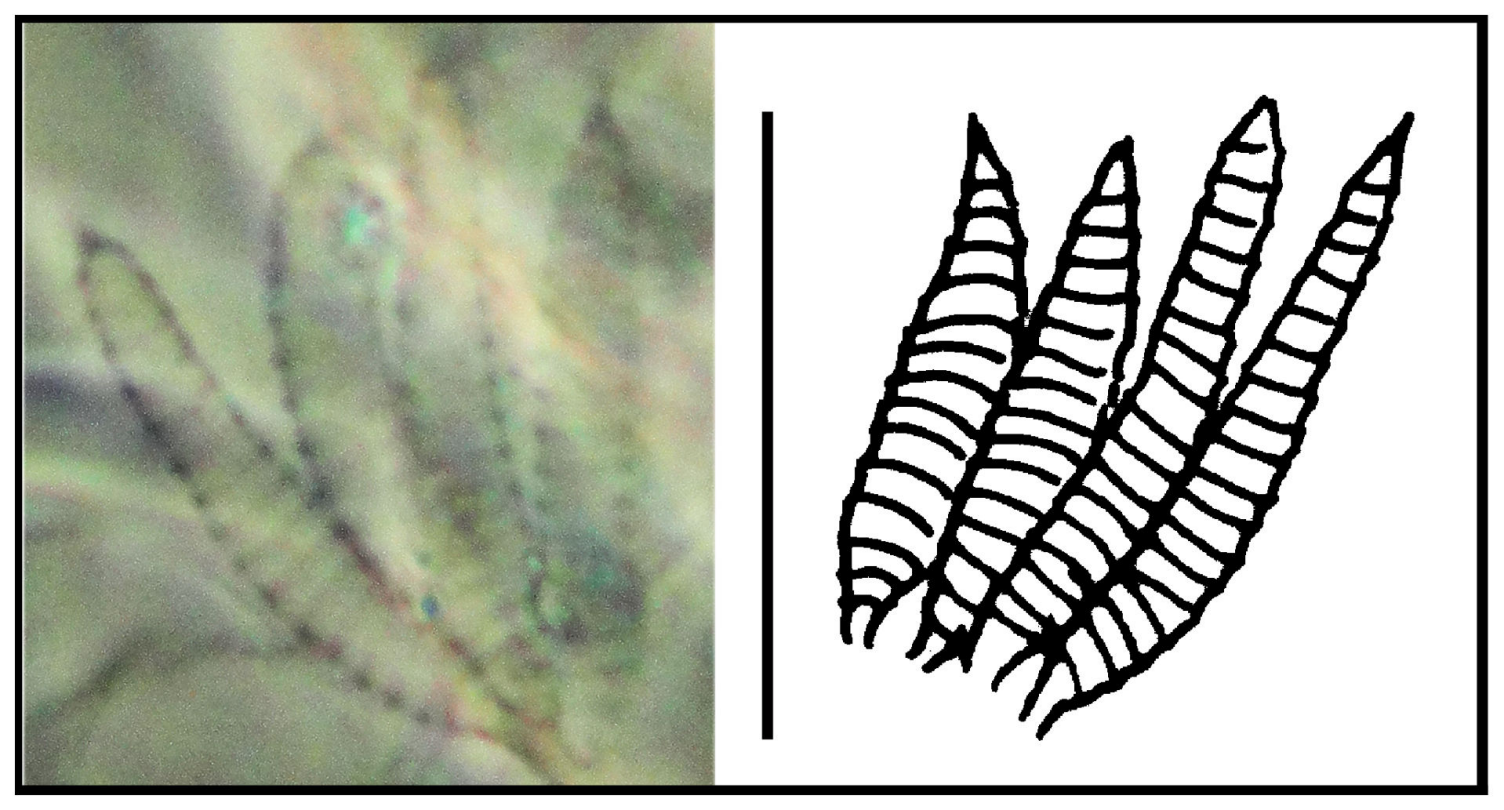
*Leptokoenenia pelada* sp. nov. Lateral organs of male. Scale bar 20 µm.

**Figure 3:**
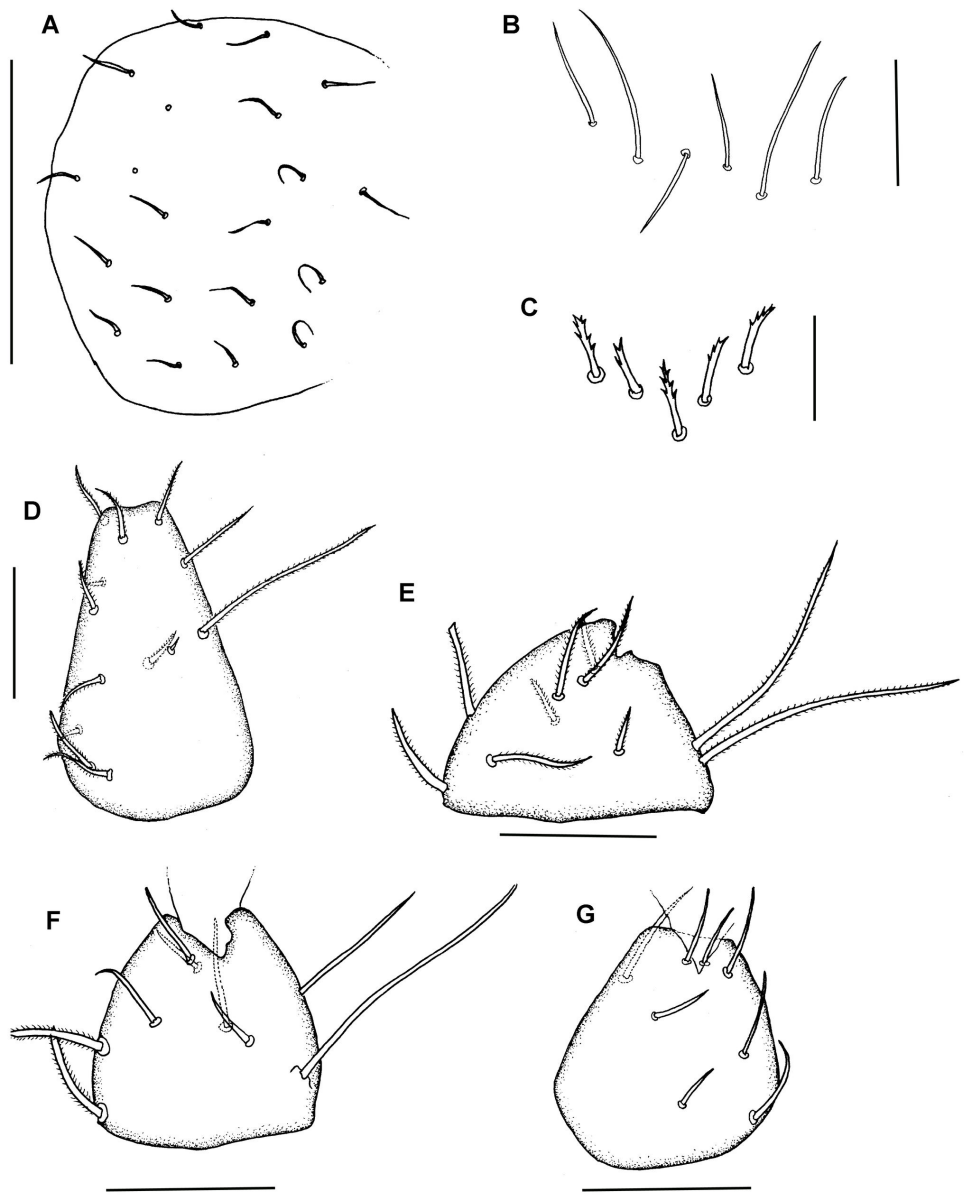
*Leptokoenenia pelada* sp. nov. (A) Propeltidial chaetotaxy of male. Scale bar 150 µm, (B) Metapeltidial setae of male. Scale bar 40 µm, (C) Deutotritosternal setae of male. Scale bar 40 µm, (D-G) Coxae I - IV of female 1 (Holotype, ISLA 1936). Scale bars 40 µm.

**Figure 4:**
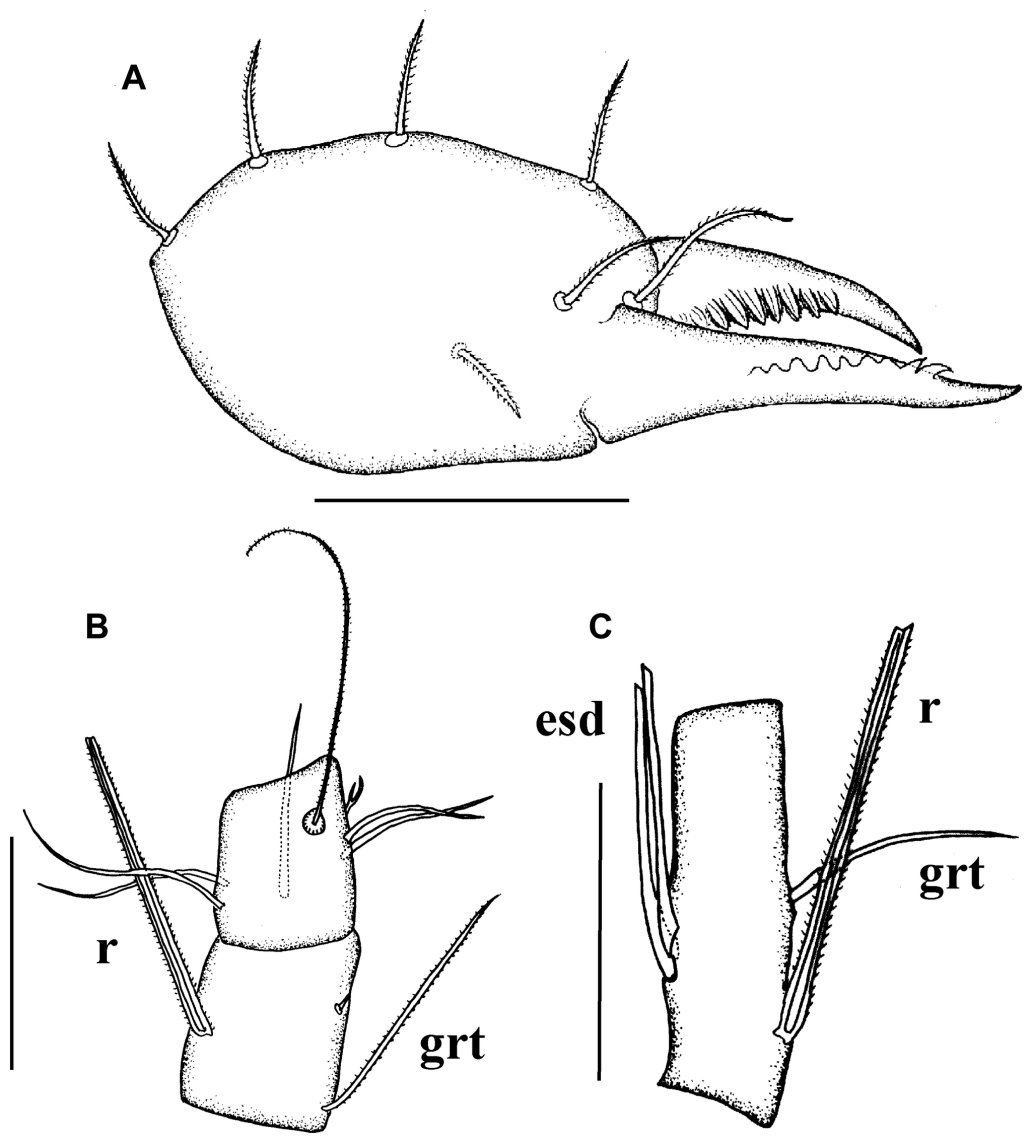
*Leptokoenenia pelada* sp. nov. (A) Chelicera of female 1(Holotype, ISLA 1936). Scale bar 60 µm, (B) Basitarsus 3 - 4 of leg I of female 1 (Holotype, ISLA 1936). Scale bar 40 µm, (C) Basitarsus of leg IV of male. Scale bar 40 µm.

**Figure 5:**
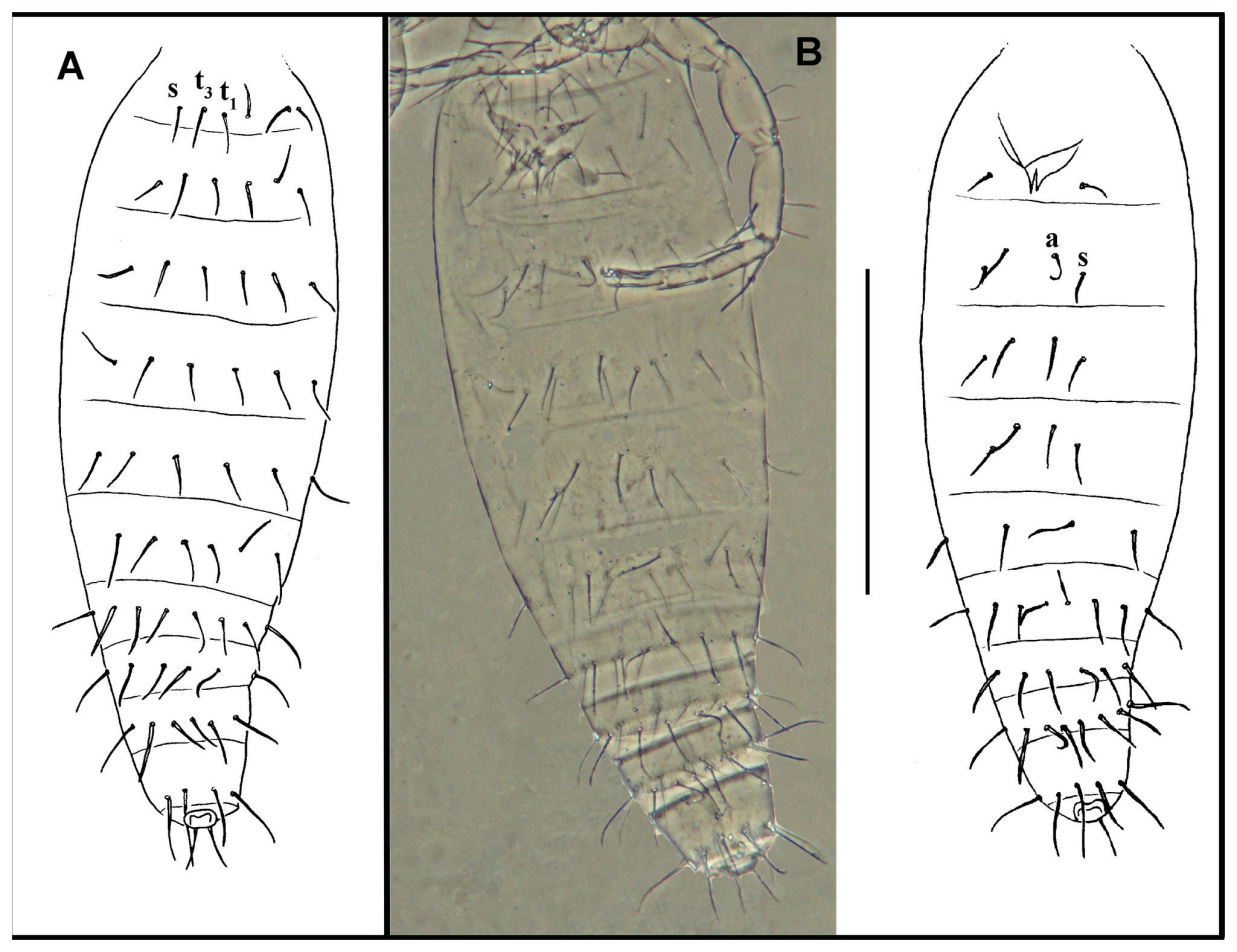
*Leptokoenenia pelada* sp. nov. (A) Opisthosoma of female 1 (Holotype, ISLA 1936), dorsal view, (B) Photograph and drawing of the opisthosoma of female 1 (Holotype, ISLA 1936), ventral view. Scale bar 300 µm.

**Figure 6:**
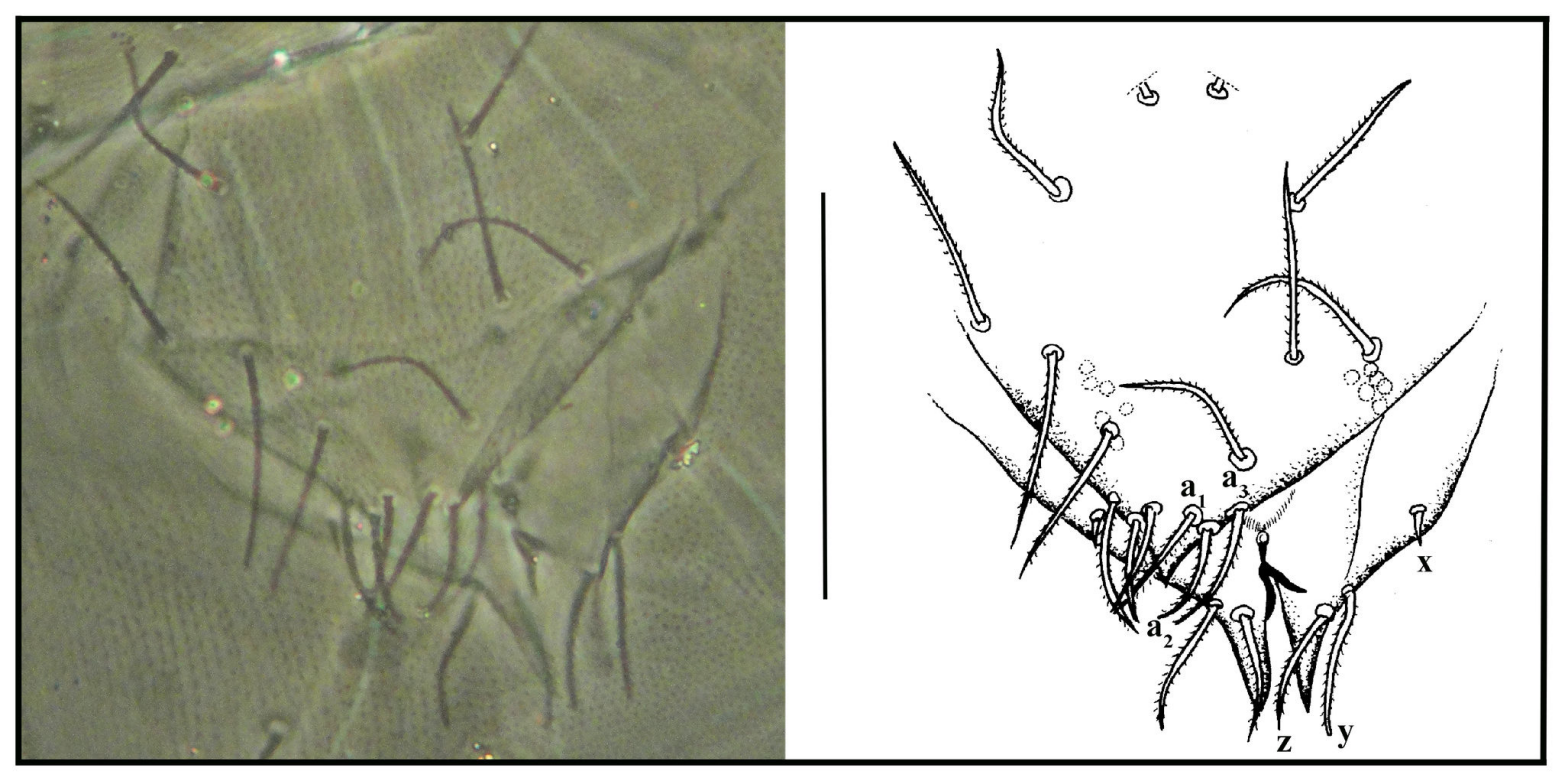
*Leptokoenenia pelada* sp. nov. Genitalia of female 1 (Holotype, ISLA 1936). Scale bar 60 µm.

**Figure 7:**
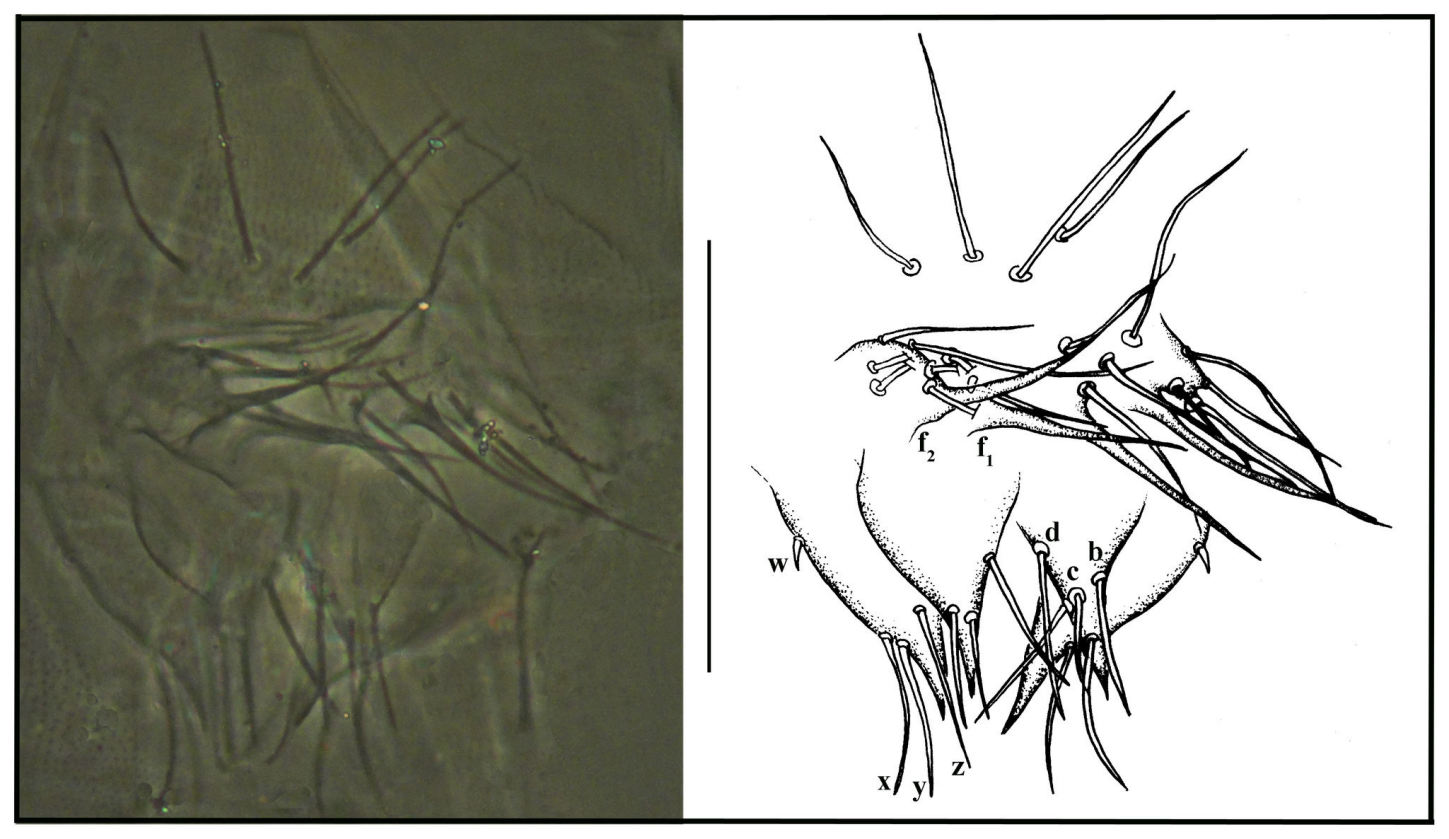
*Leptokoenenia pelada* sp. nov. Genitalia of male. Scale bar 60 µm.

**Figure 8:**
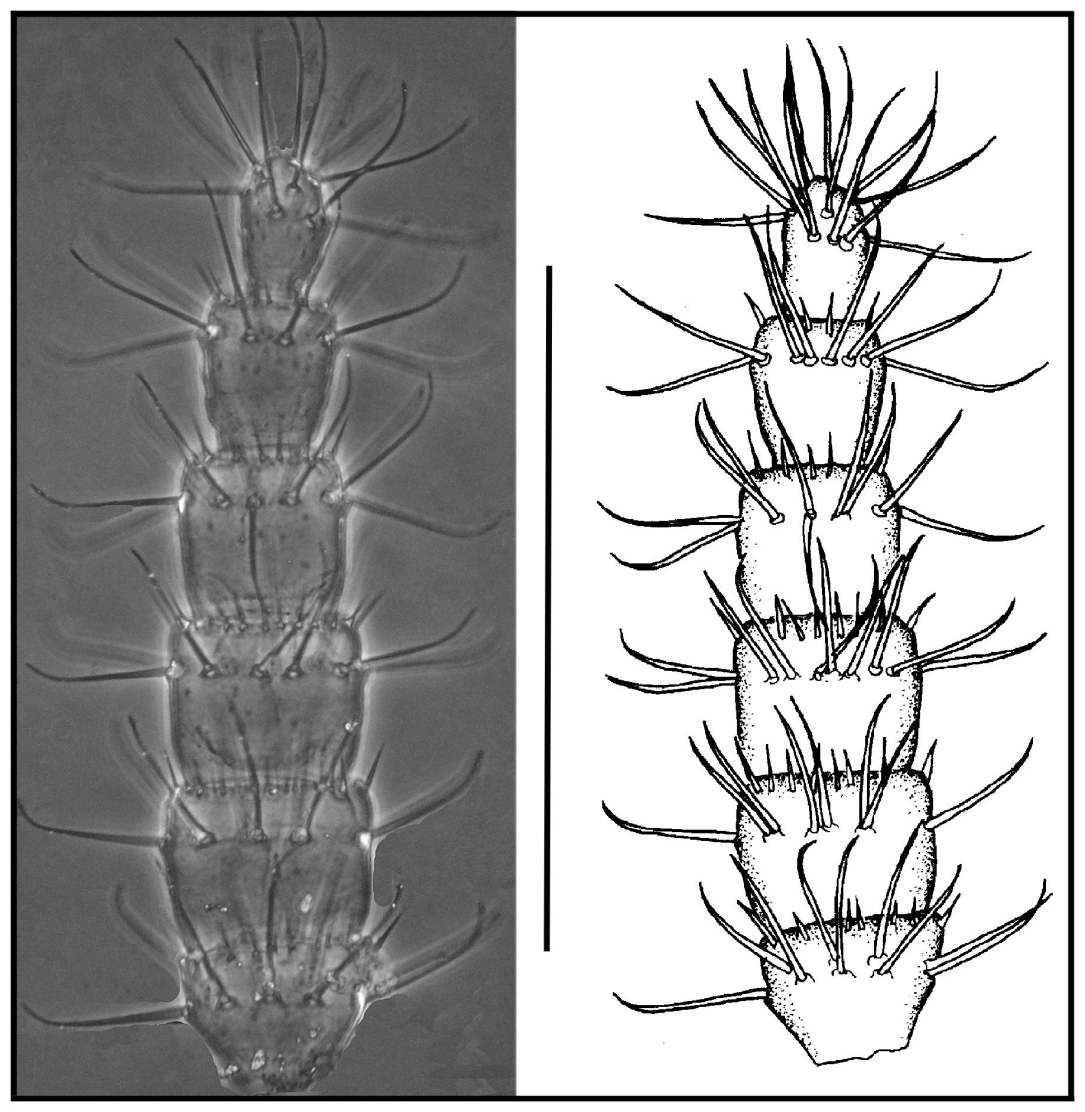
*Leptokoenenia pelada* sp. nov. Male flagellum. Scale bar 250 µm.

**Figure 9:**
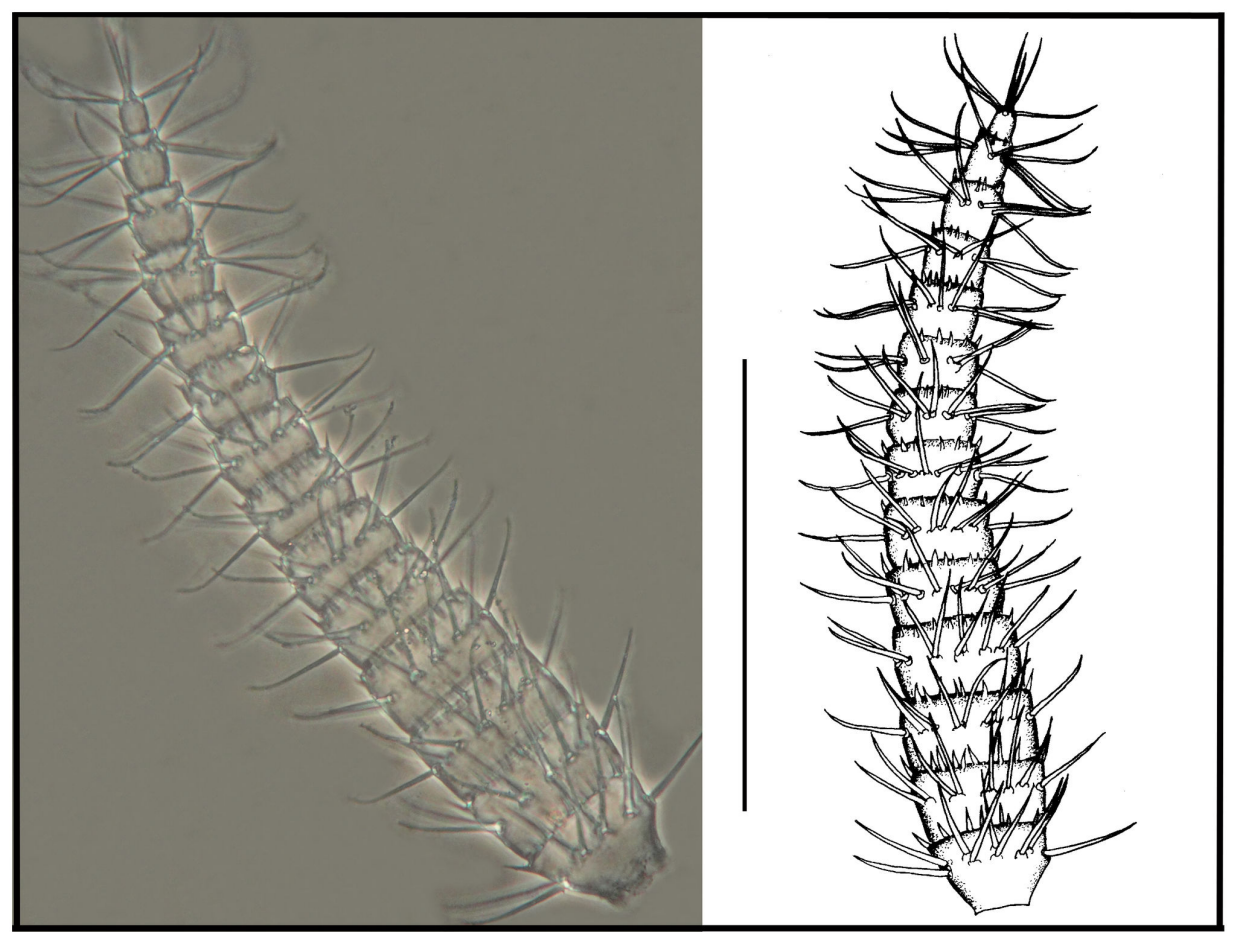
*Leptokoenenia pelada* sp. nov. Flagellum of female 1 (Holotype, ISLA 1936). Scale bar 250 µm.

#### Type material

Holotype (Female 1) (ISLA 1936), Female 2 (ISLA 1937), Male (ISLA 1938) and Juvenile (ISLA 3953) from SL42 cave (UTM 651415E/9339056N), Curionópolis, Pará, Brazil. All specimens were collected on 16/06/11; R.L. Ferreira leg. 

#### Etymology

The name *pelada*, which means “naked” refers to the reduced pubescence at the end of the abdomen. Furthermore, the cave in which the species was found is located in a group of mountains locally known as “Serra Pelada”, which means “naked mountains”, in reference to the presence of the metallophilic vegetation, that is considerably reduced (in size) when compared to the surrounding Amazon forest.

#### Diagnosis

*Leptokoenenia pelada* displays the following combination of characters: frontal organ with rounded branches, presence of four blades with ring-shape structure on prosomal lateral organs; four setae on basitarsus IV (2 esd, grt and r); opisthosomal sternites IV–VI with a pair of thick setae between a pair of normal setae; pubescence reduced to a circular fringe on segments IX - XI; genitalia of adult female with 8+8 setae on first lobe and 3+3 on second lobe; genitalia of adult male with 12 + 12 setae (including 2+2 relatively long fusules without median constriction) on first lobe, 3 + 3 setae on second lobe and 4 + 4 setae on third lobe. 

#### Description

*Prosoma*. Frontal organ with two rounded branches, each 1.8 - 3.2 times longer than wide (9 - 16 µm/5 µm) (Figure 1). Lateral organ with four pointed blades, 5 - 7.4 times longer than wide (16 - 22 µm/3 - 4 µm) (Figure 2). These blades present a ring-shape structure. Propeltidium with 10 + 10 setae (Figure 3A). Metapeltidium with 3 + 3 setae (t_1_, t_2_, t_3_), median setae longest (29 - 34 µm, 47 - 52 µm and 30 - 34 µm) (Figure 3B). Deutotritosternum with five setae in V-shaped arrangement (Figure 3C). 

 Coxal chaetotaxy: coxa I with 13 setae, coxa II with 2 thick and 8 normal setae, coxa III with 2 thick and 7 normal setae and coxa IV with 8 normal setae (Figure 3D - G). 

Chelicerae with 9 teeth on each finger; four dorsal setae, one ventral seta and two setae inserted in the middle of the second segment. The third segment (movable finger) is longer than the second segment (fixed segment) (Figure 4A).

 Leg I: basitarsus 3 short, 1.3 - 1.5 times longer than wide, with three setae (grt 47.5 - 50 µm; r 52.5 - 57.5 µm). Seta r longer than segment (35 - 37.5 µm/52.5 - 57.5 µm, t/r = 0.6 - 0.7), inserted in proximal half and surpassing hind edge (27.5 - 32.5 µm/12.5 µm, s/er = 2.2 - 2.6) (Figure 4B). With 7 trichobothria in usual arrangement and 7 forked setae: 3 on ta3 and 1 on ta2, bta4, bta2 and bta1 each. Leg IV: basitarsus short, 3 - 3.8 times longer than wide, with 4 setae (2 esd, grt and r) (Figure 4C), bta/ti 0.69 - 0.71. Length of stiff seta r similar to the tergal edge of article (52.5 - 57.5 µm/55 µm, t/r = 0.95 - 1) and inserted in proximal half (52.5 - 57.5 µm /7.5 - 10 µm, t/er = 5.5 - 7).

#####  Opisthosoma

elongated, without sudden narrowing in posterior region (length 670 - 685 µm). Tergites II–VI with 3 + 3 setae each, 2 pairs of tergal setae (t_1_, t_3_) between both slender setae (s) (Figure 5A). Sternite III with 1 + 1 setae. Sternites IV-VI with 2 +2 setae, a pair of thick setae (a) between both slender setae (s). A pair of glandular pores could be observed between the thick setae in females. Segments VII – XI with 8 - 10, 14 - 16, 12 - 13, 13 - 14 and 9 setae, respectively (Figure 5B). The pubescence of segments IX–XI is reduced on the ventral surface compared to other body parts and practically absent on the dorsal surface, except for a circular row of hair-like structures. The fringe is inserted along the line of the setal bases on IX and X, and caudal to this line on XI. The flagellum is connected to segment XI via an intermediate ring having 2 short dorsal and 2 long ventral setae. 

#####  Female genitalia

First lobe with 8 + 8 setae in 4 transverse rows, 3 sternal 2 + 2, 2 + 2, 1 + 1 and distal 3 + 3, of which a_1_, a_2_, a_3_ measure 15 - 16 µm, 14 - 15 µm and 21 µm, respectively. Second lobe with 3 + 3 setae (x, y, z), measuring 4 µm, 21 µm and 19 µm, respectively; six pairs of glandular orifices (Figure 6). 

#####  Male genitalia

First lobe with 12 + 12 setae (including 2+2 fusules). Fusules with the same length (34 µm), without median constriction, with its caliber progressively decreasing from the base to the apex. Second lobe subtriangular, with a simple and sharp apex (without bifurcation), with 3 + 3 setae (***a,b,c***). Third lobe also in a subtriangular form, well developed, with 4 + 4 setae (**w, *x,y,z***), with a large, sharp and simple acute apical region (Figure 7). 

#####  Male flagellum

Shorter than opisthosoma length (total length 322.5 µm), with six short and wide segments. First three segments are wider than long and the last two segments are slightly longer than wide; the fourth segment is as long as wide. All segments, except the last, present structures similar to a crown of thorns around the extremity. Segments I-VI with 8, 9, 10, 11, 9 and 12 setae, respectively, inserted in their distal half (Figure 8).

#####  Female flagellum

Shorter than opisthosoma length (total length 432.5 - 455 µm), with 14 short and wide segments. Except the last two segments, segments are wider than long. All segments, except the last, present structures similar to a crown of thorns around the extremity. Segments I - IV with 9 setae each; segments V - VII with 8 setae each; segment VIII with 9 setae; segment IX with 7 setae; segments X - XIII with 8 setae each and last segment with 6 apical setae. The setae in segments I - X are inserted approximately in the middle of the segment and in segments XI – XIII they are inserted in the distal half (Figure 9).

 Description of juvenile female (larvae B according to Condé 1996). Lateral organs with four blades, the longest 3.8 times longer than wide (19 μm/5 μm). Fingers of chelicerae each with 9 teeth. Chaetotaxy of propeltidium and deutotritosternum complete. Chaetotaxy of metapeltidium similar to the adult, with *t*_*1*_, *t*_*2*_ and *t*_*3*_ measuring 32, 51 and 33 μm, respectively. Coxal chaetotaxy: coxa I with 12 setae, coxae II - III with 2 thick and 7 normal setae each and coxa IV with 8 normal setae. Trichobothria and forked seta as in adults. Basitarsus 3 and 4 of leg I and basitarsus of leg IV similar to the adult. Sternite III with 1 + 1 setae. Chaetotaxy of sternites IV - VI and tergites II–VI similar to the adults. Segments VII – XI with 10, 14, 14, 14 and 9 setae, respectively. The pubescence of segments IX–XI is similar to the adults. Primordia of genital lobes developed on segments II but unfortunately it was not possible to determine the number and position of the setae due to damage during slide mounting. Flagellum with 14 short and wide segments (total length 455 µm), similar to the female adults. Segments I - IV with 9 setae each; segments V and VI with 8 setae each; segments VII and VIII with 9 setae each; segments IX - XIII with 8 setae each and last segment with 7 apical setae. The setae in segments I - XI are inserted approximately in the middle of the segment and in segments XII and XIII they are inserted in the distal half.

#### Leptokoenenia thalassophobica sp. nov.

**(urn:lsid:zoobank.org:act:AD0AF19C-2F63-4434-A83F-0618E8F39E0D**)

 (Figures 10 - 13)

**Figure 10:**
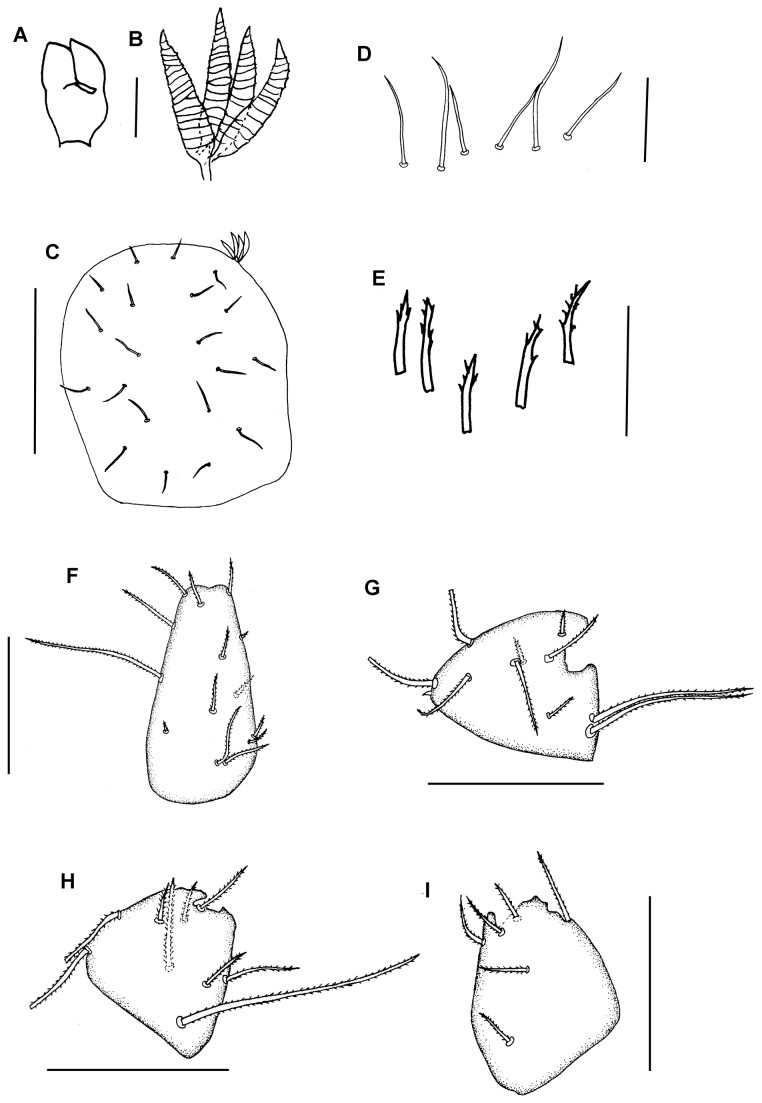
*Leptokoenenia thalassophobica* sp. nov. (A) Frontal organ of male 2. Scale bar 10 µm, (B) Lateral organs of female 1. Scale bar 20 µm, (C) Propeltidial chaetotaxy of female 1. Scale bar 150 µm, (D) Metapeltidial setae of female 1. Scale bar 40 µm, (E) Deutotritosternal setae of female 1. Scale bar 20 µm, (F-I) Coxae I - IV of female 1. Scale bars 60 µm.

**Figure 11:**
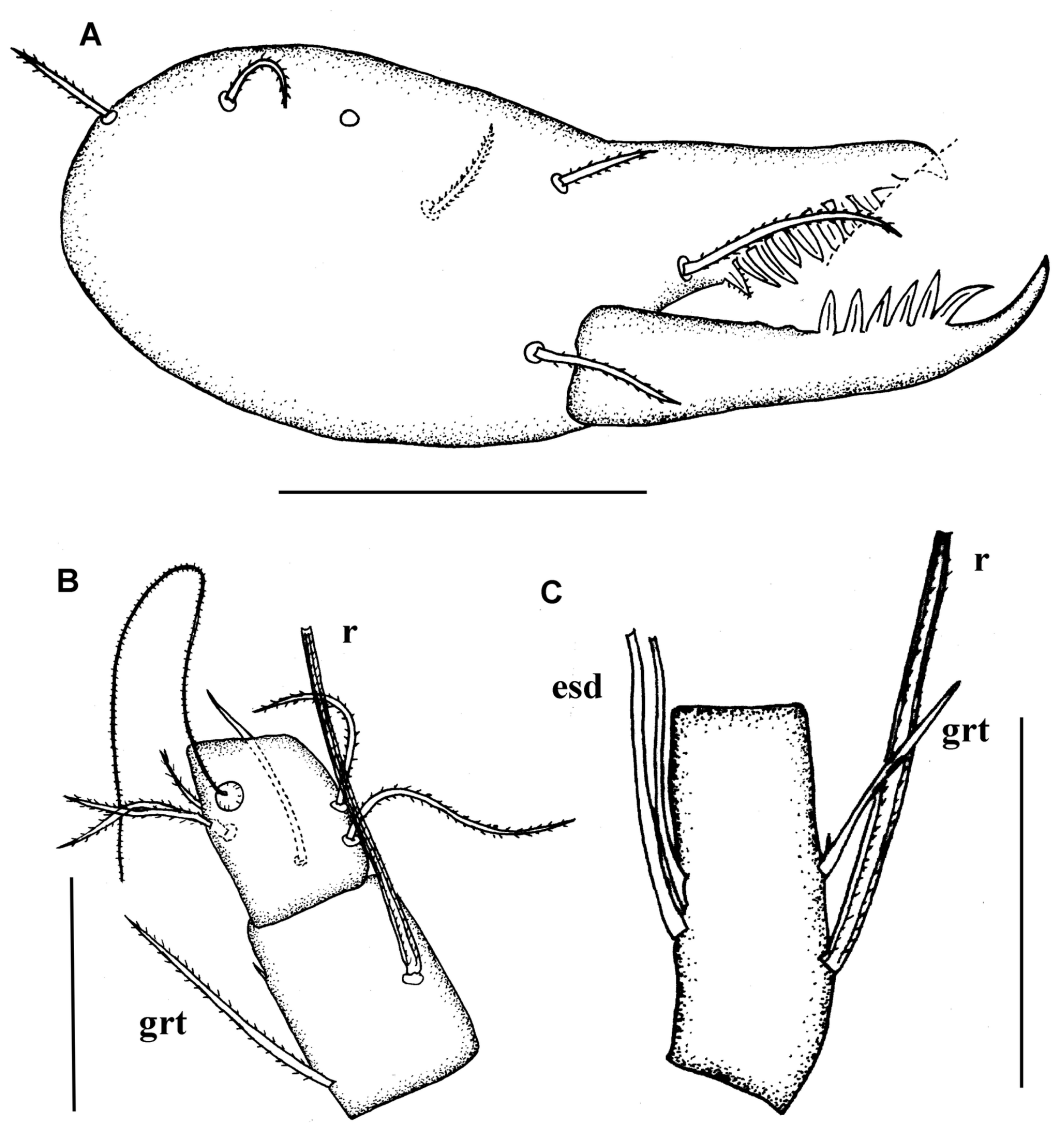
*Leptokoenenia thalassophobica* sp. nov. (A) Chelicera of male 2. Scale bar 60 µm, (B) Basitarsus 3 - 4 of leg I of male 2. Scale bar 40 µm, (C) Basitarsus of leg IV of male 1 (Holotype, ISLA 1939). Scale bar 40 µm.

**Figure 12:**
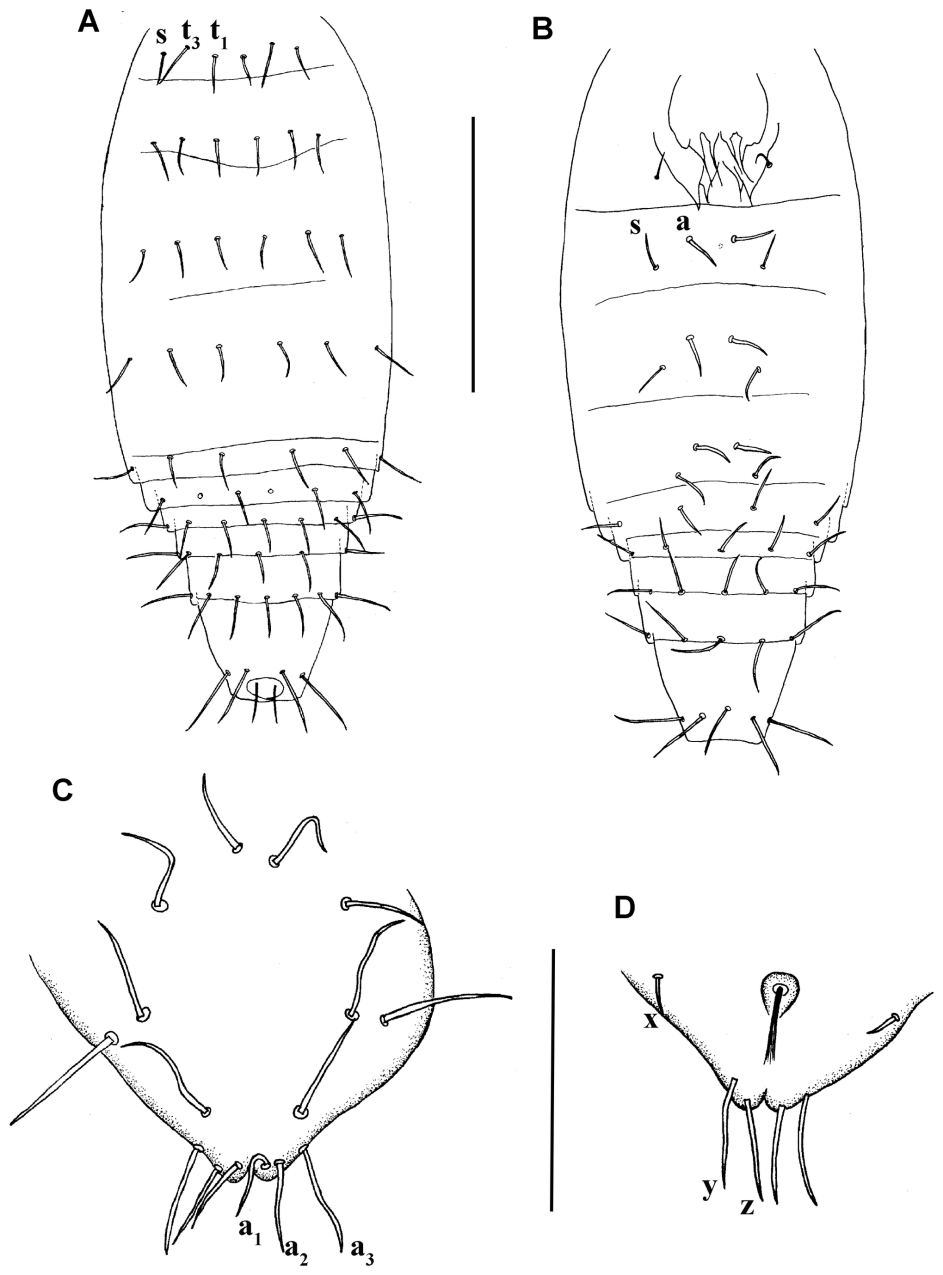
*Leptokoenenia thalassophobica* sp. nov. (A) Opisthosoma of male 1 (Holotype, ISLA 1939), dorsal view, (B) Opisthosoma of female 1, ventral view. Scale bar 400 µm, (C) First lobe of genitalia of female 2, (D) Second lobe of genitalia of female 2. Scale bar 60 µm.

**Figure 13:**
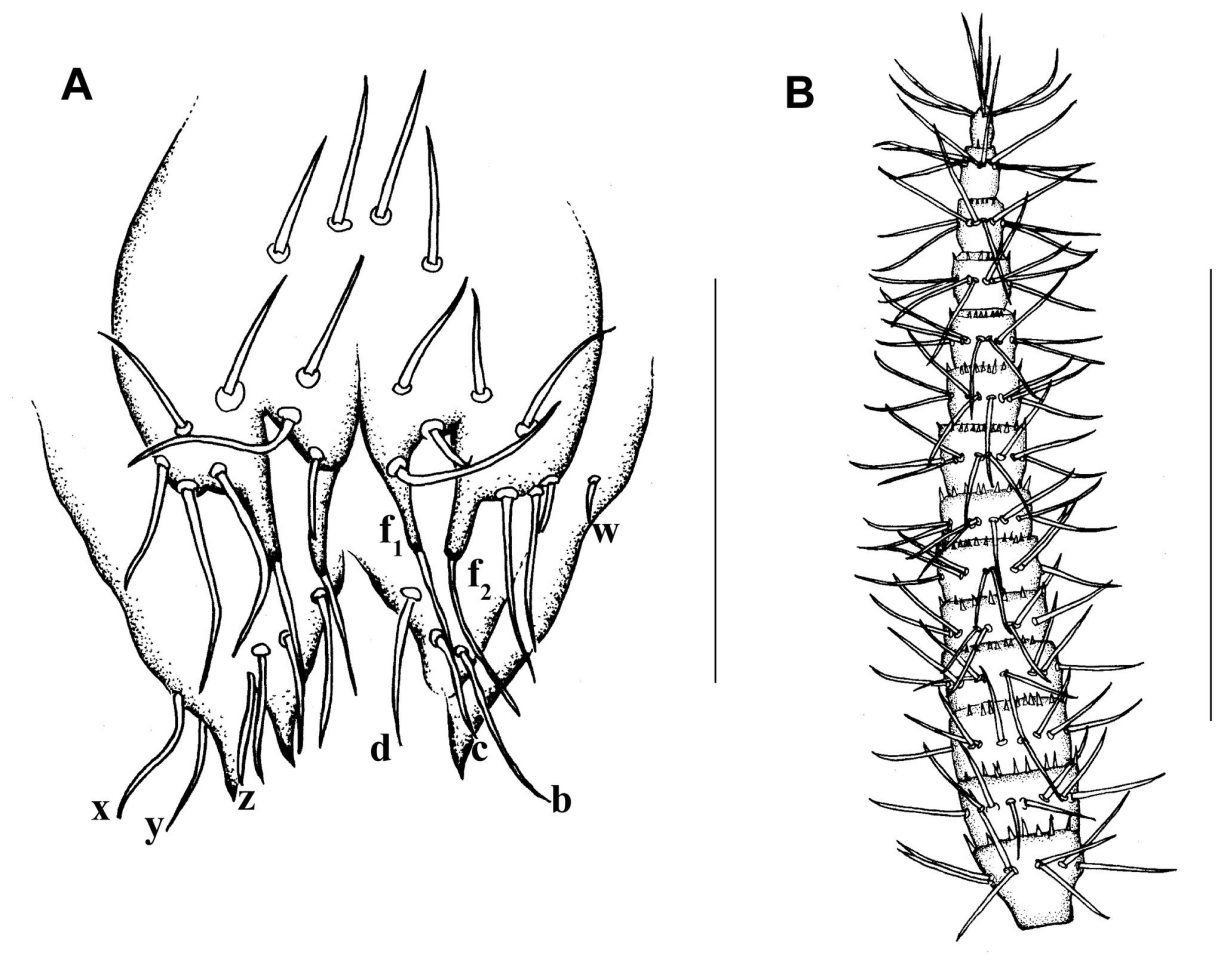
*Leptokoenenia thalassophobica* sp. nov. (A) Genitalia of male 1 (Holotype, ISLA 1939). Scale bar 60 µm, (B) Flagellum of female 1. Scale bar 250 µm.

#### Type material

Holotype (Male 1) (ISLA 1939) and Female 1 (ISLA 1940) from GEM 1769 cave (UTM 595701E/9321709N), Parauapebas, PA, Brazil. These specimens were collected on 4/11/11, M.P. Oliveira leg. Female 2 (ISLA 3954) from N4WS06 cave (UTM 0589250E/9328250N), Parauapebas, Pará, Brazil, collected on 18/11/10, R. Andrade et al. leg. Male 2 (ISLA 3955) from N4WS55 cave (UTM 0588994E/9327825N), Flona Carajás, PA, Brazil, collected on 1/06/11, R. Andrade et al. leg.

#### Etymology

The specific name is formed by a hybridism: ‘‘thalasso,’’ radical of Greek origin that means ‘‘sea’’; and ‘‘phobica’’ meaning “afraid of”. Hence, the name means “that which is afraid of the sea”, referring to the fact that the species is not found in intertidal habitats, in contrast to the previously known species *L. scurra* and *L. gerlachi*. 

#### Diagnosis

*Leptokoenenia thalassophobica* displays the following combination of characters: frontal organ with rounded branches, presence of four blades with ring-shape structure on prosomal lateral organs; four setae on basitarsus IV (2 esd, grt and r); opisthosomal sternites IV–VI with a pair of thick setae between a pair of normal setae; segments IX – XI with normal pubescence; genitalia of adult female with 8+8 setae on first lobe and 3+3 on second lobe; genitalia of adult male with 12 + 12 setae (including 2+2 relatively long fusules with median constriction) on first lobe, 3 + 3 setae on second lobe and 4 + 4 setae on third lobe.

#### Description

*Prosoma*. Frontal organ with two rounded branches, each 3.75 times longer than wide (15 µm/4 µm) (Figure 10A). Lateral organ with four pointed blades, 5.6 - 6.5 times longer than wide (17 - 26 µm/3 - 4 µm) (Figure 10B). These blades present a ring-shape structure. Propeltidium with 10 + 10 setae (Figure 10C). Metapeltidium with 3 + 3 setae (t_1_, t_2_, t_3_), median setae longest (30 µm, 50 µm and 35 - 40 µm) (Figure 10D). Deutotritosternum with five setae in V-shaped arrangement (Figure 10E).

 Coxal chaetotaxy: coxa I with 14 setae (11 in male II), coxa II with 2 thick and 8 normal setae (7 normal setae in male II), coxa III with 2 thick and 7 normal setae and coxa IV with 6 normal setae (8 in male II) (Figure 10F - I). 

 Chelicerae with at least 8 teeth on each finger; five dorsal setae, one ventral seta and one seta inserted near the teeth on the second segment. The third segment (movable finger) is longer than the second segment (fixed segment) (Figure 11A).

 Leg I: basitarsus 3 short, 1.3 - 1.6 times longer than wide, with three setae (grt 42.5 - 52.5 µm; r 55 - 57.5 µm). Seta r longer than segment (30 - 37.5 µm/55 - 57.5 µm, t/r = 0.56 - 0.68), inserted in the middle of the segment and surpassing hind edge (25 - 32.5 µm/12.5 - 17.5 µm, s/er = 1.85 - 2.1) (Figure 11B). With 7 trichobothria in usual arrangement and 7 forked setae: 3 on ta3 and 1 on ta2, bta4, bta2 and bta1 each. Leg IV: basitarsus short, 1.7 - 4.2 times longer than wide, with 4 setae (2 esd, grt and r) (Figure 11C), bta/ti 0.61 - 0.68. Stiff seta r 0.8 times shorter than tergal edge of article (40 - 55 µm/45 - 62.5 µm, t/r = 0.8) and inserted in proximal half (40 - 55 µm /12 - 15 µm, t/er = 3.1 - 4).

#####  Opisthosoma

elongated, without sudden narrowing in posterior region (length 480 -555 μm). Tergites II–VI with 3 + 3 setae each, 2 pairs of tergal setae (t_1_, t_3_) between both slender setae (s) (Figure 12A). Sternite III with 1 + 1 setae. Sternites IV-VI with 2 +2 setae, a pair of thick setae (a) between both slender setae (s). A pair of glandular pores could be observed between the thick setae in the sternite IV of Male 1. However, due to the artifacts in the slides, it was not possible to observe if there are pores in the other segments or in the opisthosoma of the other specimens. Segments VII – XI with 10, 12, 11 - 12, 12 and 9 setae, respectively (Figure 12B). The pubescence of segments IX- XI is present.

#####  Female genitalia

First lobe with 8 + 8 setae in 4 transverse rows, 3 sternal 2 + 2, 2 + 2, 1 + 1 and distal 3 + 3, of which a_1_, a_2_, a_3_ measure 17 µm, 19 µm and 24 µm, respectively (Figure 12C). Second lobe with 3 + 3 setae (x, y, z), measuring 7 - 8 µm, 24 µm and 22 µm, respectively (Figure 12D). 

#####  Male genitalia

First lobe with 12 + 12 setae (including 2+2 fusules). Fusules of different length (f_1_ = 22 µm; f_2_ = 35), with their caliber progressively decreasing from the base to the apex. Second lobe subtriangular, with a simple and sharp apex (without bifurcation), with 3 + 3 setae (***a,b,c***). Third lobe also in a subtriangular form, well developed, with 4 + 4 setae (**w, *x,y,z***), with a large, sharp and simple acute apical region (Figure 13A). 

#####  Female flagellum

Shorter than opisthosoma length (total length 440 µm), with 14 short and wide segments. Segments I - X wider than long and segments XI – XIV longer than wide. All segments, except the last, present structures similar to a crown of thorns around the extremity. Segment I with 9 setae; segments II and III with 8 setae each; segment IV with 9 setae; segments V - XIII with 8 setae each and last segment with 7 apical setae. The setae in segment I are inserted in the proximal half, in the segments II – XII they are inserted approximately in the middle of the segment and in the segment XIII they are inserted in the distal half (Figure 13B).

Unfortunately, the male flagellum was lost during collection.

Morphometric data are given in Table 1. 

**Table 1:** Measurements (µm) of selected body parts of the specimens of *Leptokoenenia pelada* sp. nov. and *Leptokoenenia thalassophobica* sp. nov.

	*Leptokoenenia pelada*				*Leptokoenenia thalassophobica*			
Body part	Male	Female 1	Female 2	Juvenile	Male 1	Male 2	Female 1	Female 2
L	1005	1020	1015	970	715	800		835
B	188	195	187.5	210	175	212.5	220	205
Pti	70	72.5	70	67.5	60	70	75	77.5
Pbta1	17.5	17.5	17.5	17.5	15	17.5	17.5	17.5
Pbta2	25	25	25	22.5	22.5	22.5	25	25
Pta1	17.5	15	15	15	15	15	15	17.5
Pta2	20	22.5	25	22.5	20	20	20	20
Pta3	45	42.5	40	37.5	30	37.5	37.5	40
Iti	70	70	67.5	67.5	55	62.5	67.5	65
Ibta1+2	47.5	45	42.5	45	40	42.5	42.5	45
Ibta3	37.5	35	35	35	30	32.5	37.5	37.5
Ibta4	32.5	30	30	30	25	30	30	30
Ita1	20	17.5	15	17.5	15	17.5	17.5	17.5
Ita2	22.5	22.5	20	17.5	17.5	20	20	20
Ita3	72.5	72.5	72.5	72.5	55	72.5	72.5	77.5
IVti	80	80	77.5	80	65	76	77.5	80
IVbta	57.5	55	52.5	55	40	50	47.5	55
IVta1	32.5	32.5	32.5	32.5	25	30	30	31
IVta2	40	42.5	40	40	27.5	40	35	36
*A*	15	15	17.5	15	15	17.5	15	13
*er*	10	10	7.5	12.5	12.5	12.5	15	12
*grt*	32.5	32.5	30	30	-	30	30	-
*R*	55	55	55	60	45	62.5	57.5	-
*t/r*	1	1	0.95	0.91	0.88	0.8	0.82	-
*t/er*	5.75	5.5	7	4.4	3.2	4	3.1	-
B/btaIV	3.26	3.5	3.4	3.8	4.37	4.25	4.6	4
btaIV/tiIV	0.71	0.69	0.7	0.68	0.61	0.65	0.61	0.68
FI	50	45	-	45	-	-	48	-
FII	52.5	37.5	-	35	-	-	35	-
FIII	60	40	-	37.5	-	-	35	-
FIV	57.5	37.5	-	35	-	-	32	-
FV	52.5	32.5	-	30	-	-	27	-
FVI	50	32.5	-	32.5	-	-	30	-
FVII	-	27.5	-	32.5	-	-	29	-
FVIII	-	27.5	-	35	-	-	32	-
FIX	-	25	-	30	-	-	31	-
FX	-	27.5	-	32.5	-	-	28	-
FXI	-	27.5	-	30	-	-	25	-
FXII	-	25	-	30	-	-	25	-
FXIII	-	27.5	-	30	-	-	25	-
FXIV	-	20	-	20	-	-	19	-

#### *Leptokoenenia gallii* (Christian 2009)

New Combination

*Eukoenenia gallii* Christian [19]: 59 - 68; Christian et al. [20]: 829 - 834.

Material examined: two females from Italy, Liguria, Savona Province, Bergeggi (44°15′27″N, 8°26′35″E); 5 July 2007, leg. M. Capurro, D. Duradoni & *L. Galli*, 2007 - 2008.

Diagnosis: see Christian [19]

Description: see Christian[19] 

Remarks: the establishment of the new combination is justified by the fact that *Eukoenenia gallii* present the diagnostic features of the genus *Leptokoenenia*. According to Christian et al. [20], the opisthosoma of this species lacks the sudden constriction after the eighth segment observed in most species of the genus *Eukoenenia*, resembling in this respect, the species of the genus *Leptokoenenia*. This species, like the Brazilian species, is not associated with coastal environments, being found in soil under *Quercus suber* trees, near the coastal region in northwest Italy.

## Discussion

The two new species of the genus *Leptokoenenia* are morphologically very similar, sharing most of the features of taxonomic significance. These, in turn, can be distinguished by the shape of the fusules and the first lobe of the male genitalia, the spermatheca of the female genitalia and the pubescence of the IX-XI abdominal segments which is reduced in *L. pelada* and "normal" in *L. thalassophobica*. It is also important to note that *L. thalassophobica* has the values ​​of some morphological measurements considerably lower than *L. pelada*, as can be seen in Table 1. Furthermore, there is a little variation in the insertion height of the seta r in basitarsus IV between individuals, as evidenced by the *t/er* ratio values in Table 1, being that individuals of *L. thalassophobica* have a lower ratio (3.1 - 4) as compared to *L. pelada* adults (5.5 - 7).

The structure of the lateral organs of *L. pelada* and *L. thalassophobica* is quite unusual. These organs present ring structures, whereas the other species of Palpigradi usually have a structure with irregular reticulation. In addition, the frontal organs of these two new species do not show the typical reticulation present in the majority of the species described. These organs are presumably sensorial and correspond to modified setae [21]. Histological studies would be interesting for a more detailed comparison of these structures and identification of possible functions.

The new species share some characteristics with *L. scurra*, such as the number and arrangement of the setae in the second and third segments of the chelicerae, the number of setae in bta IV, number of setae in the deutotritosternum (five in some specimens) and the rounded shape of the frontal organs. A quite remarkable feature shared by *L. scurra* and *L. pelada* is the presence of fourteen segments in the flagellum of females and six in the male flagellum. The flagellum of females of both species can be distinguished by the number of setae in each segment. Regarding the flagellum of the males, Monniot [22] does not make a detailed description of the chaetotaxy. Furthermore, the author remained in doubt if it corresponded to a flagellum under regeneration due to the smaller number of segments or if it was a secondary sexual characteristic. With the observation of this feature in *L. pelada*, it can be stated that *L. pelada* and *L. scurra* exhibit sexual dimorphism with respect to the number of segments of the flagellum.

Regarding the differences between Brazilian species and *L. scurra*, one can cite the number of blades on the lateral organs, the insertion height of the seta r in basitarsus IV, the number of setae on the first lobe of the male genitalia and the lobes of the female genitalia. According to Monniot [22], *L. scurra* features 6 + 6 setae on the second lobe, and 1 + 1 on the third lobe. However, this layout is not common in different species of Palpigradi since the third lobe, in most cases, has 4 + 4 setae. Thus, according to the previous statement, and as illustrated by the author, it is plausible to assume that *L. scurra* presents three pairs of setae in the second lobe and four pairs in the third lobe, as do the two Brazilian species.

It is also important to note that *L. pelada* has some features in common with the species *Leptokoenenia gallii*. Among the shared characteristics, we can mention the reduced pubescence in the opisthosomal segments IX-XI, the flagellum of the female formed by fourteen wide and short flagellomeres, the presence of five setae on the deutotritosternum and a short bta IV with four setae (2 esd, r and grt); the last three characteristics were also observed in *L. thalassophobica*. *Leptokoenenia gallii* differs from the two new species mainly by having lateral organs formed by three blades with reticulated structure, frontal organ formed by lanceolate blades, female genitalia with 11 + 11 setae on the first lobe and genitalia of the male with 11 + 11 setae on the first lobe and 4 + 4 on the second lobe, besides presenting lobes with different morphology. The chaetotaxy of the opisthosomal sternites and tergites is also quite different between the Brazilian species and Italian species [19].

Because the type species of the genus, *Leptokoenenia gerlachi*, was described from a larva (stage A) and none of the specimens analyzed in the description herein of the two new species are found in the same stage of development, reliable comparisons between these three species could not be made.

The values ​​of the bta/ti ratios in *L. pelada* (average 0.69) and *L. thalassophobica* (average 0.63) were higher than those observed in *L. gerlachi* (0.5) and *L. scurra* (0.49), and similar to that observed in *L. gallii* (average 0.66). This indicates that although found inside caves, the new species lack elongation of appendages. The values ​​of the B/bta ratio could not be compared because the length of propeltidium is not available in the descriptions of the two species previously known. Although the values ​​of the bta/ti and B/bta ratios found indicate that the new species have very short appendages similar to typical edaphomorphic Palpigradi, it is important to remember that these ratios are established based on the genus *Eukoenenia*. Therefore, the values ​​indicative of troglomorphisms may be different in other genera.

Moreover, considering that the average ratio for the genus is around the values ​​reported for *L. pelada*, *L. thalassophobica* and *L. gallii*, the appendages of *L. scurra* and *L. gerlachi* may have become shorter in response to the intertidal habitat in which these species occur, as suggested by Condé [23]. However, the hypothesis remains speculative since it would need a cladistic analysis to establish the plesiomorphic state of this character.

## Biological Aspects

**Figure 14:**
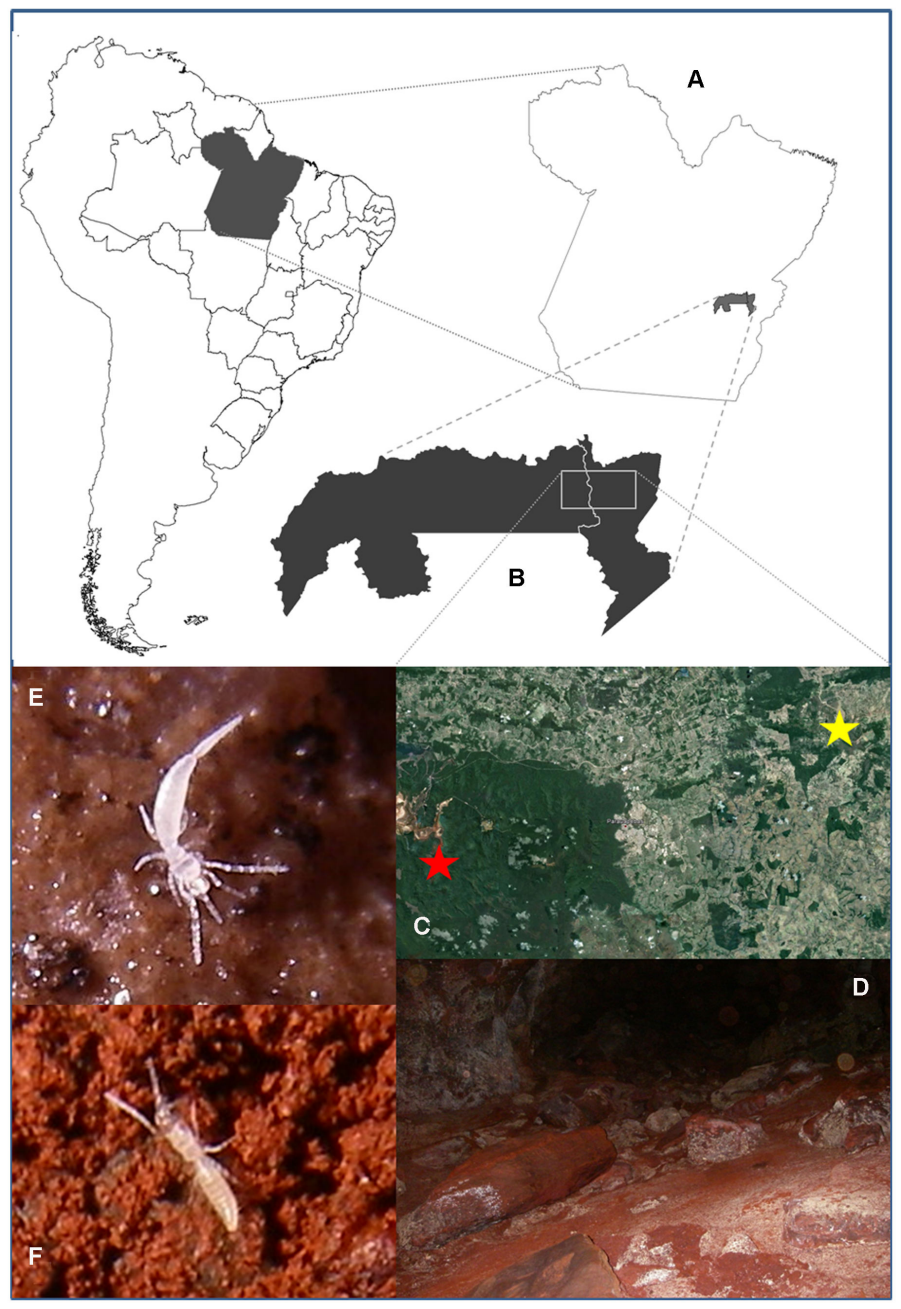
Distribution of *Leptokoenenia* species in Brazil. (A - C) Occurrence area of *Leptokoenenia pelada* (yellow star) and *L. thalassophobica* (red star), (D) Inner portion of a cave in wich specimens of *L. thalassophobica* were found, (E) Live specimen of *L. pelada*, (F) Live specimen of *L. thalassophobica*.

 The occurrence of *Leptokoenenia pelada* and *L. thalassophobica* in ferruginous caves in the Brazilian Amazon reveals that species of this genus are not exclusively intertidal and the genus is more widely distributed than previously thought. Both new species were always associated with interstitial spaces under small rocks that were commonly found in the caves´ floor (Figure 14D). They were never observed freely walking on the cave floor or surface of rocks. Living specimens can be seen in Figure 14 (E - F). A similar case was found in the millipede species of the genus *Thalassisobates* Vehoeff, 1908 in Spain: one species occurs in the littoral whereas two new species from caves*/*mesovoid shallow strata far from the coast were recently described [24]. This author gave other examples of similar habitat duality like the pseudoscorpion genus *Paraliochthonius* Beier, 1956 and the terrestrial isopod genus *Halophiloscia* Verhoeff, 1908. 

The areas where the two species described here occur are separated by approximately 60 km (Figure 14A - C). However both regions are extremely threatened by mining activity. In this context, it should be noted that type specimens are used as a tool for valuation of caves in accordance with Brazilian law. Caves that are type localities are given a high degree of relevance and this means that they can only be destroyed by preserving two other caves with similar attributes. Therefore, the description of new taxa found in Brazilian caves assists in the preservation of these fragile ecosystems.

 Brazilian cave environments have proved to be extremely favorable to the occurrence of Palpigradi [7] although many organisms of this order are also found in lapidicolous environments and in soil, unlike the temperate regions, where species are preferentially cave dwelling. Although only a single species of this order has been formally described for the Amazon [6], preliminary analyses have shown that the caves in the Amazon region have a high species richness of these arachnids, including species belonging to genera for which we have had no record for the American continent (unpublished data). Thus, the study of the biodiversity of this little-known group is extremely important for the conservation of caves included in this biome.
